# Identifying people at high risk for developing sleep apnea syndrome (SAS): a cross-sectional study in a Pakistani population

**DOI:** 10.1186/1471-2377-8-50

**Published:** 2008-12-17

**Authors:** Fawad Taj, Zarmeneh Aly, Mahwash Kassi, Mansoor Ahmed

**Affiliations:** 1 Neurology, Department of Medicine, Aga Khan University, Karachi – 74800. Pakistan; 2 Aga Khan University, Karachi, Pakistan; 3 Medical Director, FCCP, DABSM, FAASM, Southwest Cleveland Sleep Centers, Cleveland OH-44133, USA

## Abstract

**Background:**

Obstructive Sleep Apnea (OSA) is associated with many cardiovascular and psychiatric diseases. Day-time sleepiness is a common consequence of sleep apnea and correlates with road-traffic accidents (RTA). Pakistan has a high prevalence of factors which predispose an individual to OSA and death from RTAs are a huge burden. However there is a dearth of prevalence studies in this regard. We aim to understand local relevance of the disease and estimate the prevalence of individuals high-risk for OSA.

**Methods:**

This cross-sectional survey was conducted among 450 individuals at Aga Khan University Hospital (AKUH), which is a tertiary care teaching hospital in Pakistan. We used the BQ as our measurement tool. Based on the responses, participants were grouped into high or low-risk for OSA.

**Results:**

Our study sample size was 418 with 63.2% males. Mean age of our study population was 30.4 SD +/- 12.3 years; and mean BMI was 23.2 SD +/- 5 kg/m2. Out of the total sample size 24.9% reported snoring and there were twice as many males who snored as compared to females. Forty-five individuals reported that they had nodded off to sleep while driving at least once in their lifetime. On the other hand, the highest proportion of high risk individuals 47.6% was found in the age group 60 or above. The overall prevalence of individuals who were high risk for sleep apnea was 10%.

**Conclusion:**

A significant proportion of the population is at high-risk for OSA. Our study shows that despite low BMI and favorable craniofacial anatomy sleep apnea is still a locally relevant disease. Given the local relevance of OSAS, it is important to increase awareness among general population but more importantly among physicians of the developing countries, like Pakistan, about common clinical features and pertinent risk factors and complications of OSAS.

## Background

Sleep apnea is a breathing disorder characterized by snoring, breathing pauses and excessive sleepiness while awake. It is a potentially life threatening condition that is far more common than generally understood. It is associated with a number of cardiovascular, metabolic and neuro-cognitive complications. These complications include hypertension [1,2], coronary artery disease [3], congestive heart failure [4], cerebrovascular disease [5], atrial fibrillation[6], glucose intolerance [7], impotence [8] and type-2 diabetes [9]. It is also related it to increased cardiovascular mortality [10] and a high prevalence of psychiatric co-morbid conditions such as depression, anxiety, posttraumatic stress disorder, psychosis, and bipolar disorders [11]. Also it correlates with road traffic accidents involving drivers who fall asleep while driving [12].

Published data from around the world provide information regarding prevalence of Obstructive Sleep Apnea Syndrome (OSAS) the in general population. However, while comparing data from these studies, differences among methodologies and diagnostic criteria used, should be noted. Many studies used questionnaires to determine sleep apnea or its risk, where as many relied on overnight polysomnography (PSG) studies, which is the gold standard technique for sleep apnea diagnosis. The National Sleep Foundation poll, using the Berlin questionnaire, found that 26% of adults of a representative sample of the US population was at high risk for OSAS [13].

The OSAS prevalence data from Asia is scarce, however, it is reported that prevalence ranges from 2.1 to 7.5%; with a male-to-female ratio of 2:1 [14,15]. In India the prevalence of obstructive sleep apnea syndrome has been reported to be 7.5% [16]. Despite the high prevalence of OSAS and associated complications, majority of the patients remain undiagnosed. For example, in US 93% of women and 82% of men with moderate to severe OSA are clinically undiagnosed [17]. It is perhaps not unlikely that in less developed world, like Pakistan, an even greater proportion of patients remain undiagnosed.

Systematic studies done on the Pakistani population investigating the prevalence of OSAS are lacking. A study done by Haqqee et al, showed a high prevalence of snoring (46%); frequency of snoring with apnea was reported to be 7% and; snoring with apnea and excessive daytime sleepiness was 3% [18]. Haqqee et al, however did not use a structured and/or a standardized questionnaire or an overnight PSG. Another study recently done on seminar participants by the authors, Taj et al [19], reports that significant proportion of population is at risk for OSAS. Ironically, most physicians in Pakistan are unaware of the clinical features and common associations of OSAS with 18% physicians treating sleep disturbances with sedatives only [20].

The purpose of this cross-sectional study is to perform a survey of a Pakistani population using the standardized Berlin questionnaire [21] and estimate the prevalence of individuals who are at high risk for OSAS. The questionnaire uses clinical symptoms such as snoring, day time fatigue and hypertension to classify subjects as being either high risk or low risk for OSAS, with 86% sensitivity, 77% specificity, a positive predictive value of 0.89, and a likelihood ratio of 3.79. There is only one study that has so far been done in the Pakistani population to assess the OSAS prevalence which has employed the use of the Berlin Questionnaire, but it was restricted to seminar participants only [19]. We also aim to assess age, BMI and/or gender as indicators of risk for OSAS.

## Methods

### Study setting and population

The study was conducted at the Aga Khan University Hospital (AKUH), Karachi, Pakistan. It is a 542 beds tertiary care hospital and one of the leading educational institutes in the country. AKUH is the first hospital in Pakistan to be awarded the prestigious Joint Commission International Accreditation (JCIA) for practicing the highest internationally recognized quality standards in health care. It has a large, broad based and busy out-patient service. The out-patient services range from pre-employment clinics and executive check-up clinics to specialty clinics, such as stroke, hepatic diseases, hand-trauma etc. Patients comprise of all walks of life, from general population, come to this tertiary care hospital from different parts of the country. In accordance with the cultural norms, the patient scheduled for an appointment with the consultant physician, is usually accompanied by at least one healthy attendant, relative or friend.

This cross-sectional survey was conducted on individuals who had come to the hospital as attendants or visitors of the patients. The sample was collected via convenience sampling from out-patient clinics and community health center of the hospital. The study was in accordance to the Helsinki declaration and the ethics review committee at the Aga Khan University approved it. Patient information, including names that could link an individual to the data was not recorded to assure confidentiality. An informed verbal consent was obtained from each participant and documented before the administration of the questionnaire.

### Inclusion criteria

All healthy individuals, above 18 years of age, who were accompanying a patient or were visiting a friend or relative admitted in the hospital, were included. Study objectives were explained and verbal consent was obtained prior to inclusion in the survey.

### Exclusion criteria

Following individuals were excluded from the sample:

1. Any individual who was unable to read and/or understand Urdu, the national language.

2. Any individual not willing to participate in the survey.

3. Any individual who had a current illness, except hypertension.

4. Any individual who had come to the hospital for medical advice (consultation with a physician), or for a surgical procedure or help from associated medical staff.

5. Any individual who was currently in regular follow-up with any physician for an illness or was taking any prescription medication.

6. All persons associated with health care including doctors, nursing staff and medical students.

### Study Sample size

The sample population was above 18 years of age. We required a sample size of 443 subjects to fulfill the objectives of our study at a 95% confidence level. This sample size was calculated assuming a 50% prevalence of OSAS and 5% bound of error and then inflated by 15% to account for non-respondents and incomplete questionnaires.

After rounding-off the required sample, we conducted the Berlin questionnaire on a total of four hundred and fifty individuals. Thirty-two did not answer one or more questions or terminated the interview before completion. A total of 418 (94%) completed the interview (questionnaire) and were included in the final analysis.

### Questionnaire

The instrument called the Berlin questionnaire [21], that has been used to identify patients who are likely to have OSAS [15], was used as the screening tool. It has been previously employed in similar primary care settings [21,22]. This instrument inquires about snoring behavior, daytime sleepiness, obesity, and hypertension as symptoms which help in classifying patients as being either high risk or low risk for having sleep apnea syndrome. It has a sensitivity of 0.86, a specificity of 0.77, a positive predictive value of 0.89, and a likelihood ratio of 3.79. It has been validated as an authentic tool in identifying high risk patients for Sleep Apnea syndrome in many, including US [22] and Indian [23] populations. However it is not validated for our Pakistani population. For our study, we translated the questionnaire into Urdu, see additional files 1. Back-to-back method was adopted for translation of the questionnaire.

The Berlin questionnaire begins with questions about age, height, weight and sex. The questionnaire is then divided into four categories. In the first category, there is one initial question about snoring, followed by four further questions about the intensity and frequency of snoring and witnessed episodes of apneas. In the second category there were two questions related to day time fatigue and tiredness, followed by two questions about sleepiness while driving. There was only one question in the third category regarding high blood pressure. BMI was also calculated from the reported height and weight of the individual.

### Data collection

The Berlin questionnaire was administered by a group of 4th year medical students of Aga Khan University, Karachi, Pakistan. Students were given ten hours of education related to OSAS patho-physiology and its clinical features and the questionnaire. The questionnaire was discussed and students were trained to uniformly administer the questionnaire and to adopt a standardized method of interviewing.

### Data entry and statistical analysis

Data was double entered and analyzed in Statistical Package for Social Sciences 14.0 (SPSS, Inc., Chicago, IL, USA) Descriptive statistics including frequencies, means ± standard deviations (SD) were calculated. Where appropriate, chi-square was used to calculate statistical significance for qualitative variables and student t-test was used for qualitative variables. Based on the responses of the participants to the Berlin questionnaire they were grouped into either high or low risk for OSAS.

In category 1, a positive score for risk was defined as an answer in the affirmative to either of the following question: snoring with intensity "louder than talking" or very loud, snoring frequency > 3–4 times a week, snoring enough to bother other people, or witnessed apneas during sleep > 3–4 times per week.

In category 2, a positive score was defined as a patient having a frequency of symptoms >3–4 times per week in two or more questions about waking time sleepiness, and/or drowsy driving.

In category 3, a positive score is given if the participant has hypertension or has BMI greater than 27 kg/m2, the cut-off for obesity adjusted for the Asian population according to the latest guidelines [24,25].

Individuals were considered high risk for OSAS, if they scored positive in two or more categories. Those who did not have symptoms or scored positive in less than two categories were placed in the low risk group.

## Results

Of the total 418 participants included in final analysis 264 (63.2%) were males and 154 (38.4%) were females. Of the 418 individuals, 42 were at high-risk for OSAS according to our instrument – Berlin Questionnaire. Hence, the prevalence estimate of individuals at high-risk for OSAS was 10%.

Mean age was 30.4 ± 12.3 yrs (31.4 ± 13.1 years for males and 28.6 ± 10.7 years for females). The mean BMI was 23.2 ± 5. Table 1 shows different characteristics of the study population and their means ± S.D. along with comparison of mean values by gender and risk-group.

**Table 1:** Characteristics mean of the study population.

**Characteristics**	**Mean ± S.D.**	**P – value**
Age *in years*	30.38 +/- 12.3	

Male	31.42 +/- 13.1	
Female	28.59 +/- 10.7	

Low risk group	28.8 +/- 10.9	< 0.001
High risk group	44.7 +/- 14.4	

Height *in meters*	1.68 +/- 0.10	

Male	1.73 +/- 0.09	
Female	1.68 +/- 0.10	

Low risk group	1.68 +/- 0.10	0.484
High risk group	1.70 +/- 0.11	

Weight *in Kilograms*	65.3 +/- 12.6	

Male	69.7 +/- 10.9	
Female	57.8 +/- 11.8	

Low risk group	63.5 +/- 10.9	< 0.001
High risk group	82.0 +/- 15.1	

BMI *in S.I units (kg/m2)*	23.2 +/- 4.99	

Male	23.6 +/- 4.81	
Female	22.7 +/- 5.26	

Low risk group	22.6 +/- 4.38	< 0.001
High risk group	28.5 +/- 6.82	

Neck circumference *in cm*	14.1 +/- 2.81	

Male	15.2 +/- 3.23	
Female	12.7 +/- 1.17	

Low risk group	13.9 +/- 2.88	< 0.001
High risk group	15.7 +/- 1.28	

We classified 223 individuals with BMI less than 23 to be normal (53.3%); 136 individuals with BMI between 23 and 27 kg/m2 to be over weight (32.5%) and 59 individuals with BMI greater than 27 to be obese (14.1%). Only 13 individuals had BMI of more than 30 of which 8 were classified as High Risk. Individuals whose weight or height was not recorded were excluded prior to data analysis and constitute those 32 individuals initially excluded.

**Figure 1:**
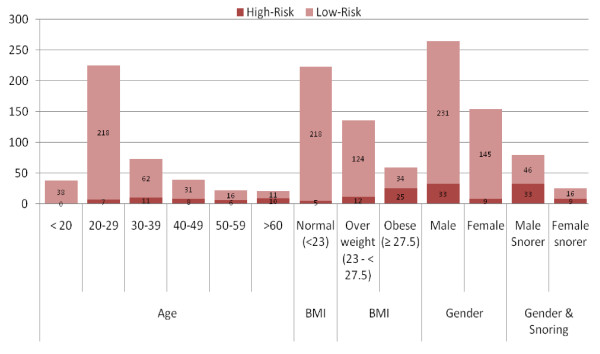
**Distribution of population age, gender, gender of snorers and BMI, according to OSAS Risk Group**.

Figure 1 shows the distribution of age, BMI and other characteristics of our study population by OSAS risk group. Most of the participants fell in 20–29 years age group (53.8%). It is interesting to note here that in the age subgroup 'less than 20' years none of the respondent was found to be high risk for OSAS. On the other hand, the highest proportion of high risk individuals 47.6% was found in the age group 60 or above. Our data shows that the risk for OSAS increased with age up to 65 years of age. Almost one-in-three participants (29%) aged 40 or more were high-risk for OSAS.

Of the 418 respondents, 104 (24.9%) reported snoring. Figure 1 shows the distribution of 79 males (18.9.2%) and 25 females (6%) snorers, with snoring being more prevalent among men as compared to women. Presence of snoring was also found to be more prevalent in individuals high-risk for OSAS (p-value < 0.0001). Of all those who snored, including both risk groups, 49% reported that they snored more than 3–4 times per week or more, 21.2% reported that their snoring was louder than talking or very loud, and 42.3% admitted that their snoring bothered others. In the same subgroup, 24% reported witnessed pauses in breathing while asleep.

Hypertension was reported by 41 individuals (7.8%). Of these 40 (97.6%) were males. Among the high-risk group 33.3% individuals had high blood pressure as compared to 7.2% of those in the low-risk group. Hypertension was found to be more prevalent in the high risk group for OSAS (p-value < 0.001).

In our study population, the prevalence of over-weight individuals (BMI ≥ 27 kg/m^2^) was significantly higher among the high-risk for OSAS group (p < 0.0001). Among the high-risk group, 5 (11.9%) had normal BMI (< 23 kg/m^2^); 12 (28.6%) were over-weight (BMI ≥ 23 -< 27 kg/m^2^; and 25 (59.5%) were obese (BMI ≥ 27 kg/m^2^). However, it is important to note here that 8 individuals (19%), among high risk, had BMI greater than 30 kg/m^2^.

According to our data, 66 individuals (15.8%) were positive for category 1; Eighty (19.1%) individuals for category 2; and 94 (22.5%) for category 3.

Table 2 shows the distribution of responses, on Berlin Questionnaire, by Risk Group.

**Table 2:** Distribution of responses to Berlin questionnaire by risk group. (n = 418)

**Questions**	**High-Risk** n (%)	**Lower-Risk** n (%)	**p-value**
Do you snore?			
Yes	42 (100)	62 (16.5)	< 0.0001
No	0 (0.00)	285 (75.8)	
Don't Know	0 (0.00)	29 (7.71)	
Snoring loudness			
Loud as breathing	16 (38.1)	47 (72.3)	< 0.0001
Loud as talking	8 (19.0)	14 (21.5)	
Louder than talking	9 (21.4)	2 (3.08)	
Very loud	9 (21.4)	2 (3.08)	
Snoring frequency			
Almost everyday	24 (57.1)	12 (18.8)	< 0.0001
3–4 times/wk	9 (21.4)	6 (9.40)	
1–2 times/wk	5 (11.9)	14 (21.9)	
1–2 times/mo	4 (9.5)	19 (29.7)	
Never or almost never	0 (0.00)	13 (20.3)	
Does your snoring bother other people?			
Yes	28 (66.7)	16 (24.2)	< 0.0001
No	14 (33.3)	40 (60.6)	
Don't Know	0 (0.00)	10 (15.2)	
How often your breathing pauses have been noticed?			
Almost everyday	9 (21.4)	6 (9.10)	< 0.0001
3–4 times/wk	1 (2.40)	1 (1.50)	
1–2 times/wk	1 (2.40)	0 (0.00)	
1–2 times/mo	0 (0.00)	7 (10.6)	
Never or almost never	31 (73.8)	52 (78.8)	
Are you tired after sleeping?			
Almost everyday	15 (35.7)	45 (12.0)	< 0.0001
3–4 times/wk	4 (9.50)	35 (9.30)	
1–2 times/wk	5 (11.9)	86 (22.9)	
1–2 times/mo	2 (4.80)	76 (20.3)	
Never or almost never	16 (38.1)	133 (35.5)	
Are you tired during wake time?			
Almost everyday	15 (35.7)	48 (12.9)	< 0.0001
3–4 times/wk	7 (16.7)	39 (10.5)	
1–2 times/wk	6 (14.3)	85 (22.8)	
1–2 times/mo	4 (9.50)	90 (24.2)	
Never or almost never	10 (23.8)	110 (29.6)	
Have you ever fallen asleep while driving?			
Yes	7 (16.7)	38 (10.3)	
No	35 (83.8)	332 (89.7)	
Do you have high blood pressure?			
Yes	14 (33.3)	27 (7.20)	< 0.0001
No	27 (64.3)	339 (90.2)	
Don't know	1 (2.40)	10 (2.70)	
Body Mass Index			
Normal (<23 kg/m2)	5 (11.9)	218 (58.0)	< 0.0001
Over weight (23 kg/m2 to less than 27.5 kg/m2)	12 (28.6)	124 (33.0)	
Obese (≥ 27.5 kg/m2)	25 (59.5)	34 (9.00)	

Tiredness and sleepiness was significantly common. Out of our total population of 418 individuals, 99 (23.7%) reported that they felt sleepy when they woke up in the morning greater than 3–4 times per week. One hundred and nine (26.1%) said that they felt tired during waking time more than 3–4 times per week. A noteworthy proportion (10.8%) of our study population reported that they had nodded off to sleep while driving at least once in their lifetime.

## Discussion and conclusion

We report the overall prevalence estimate of individuals high-risk for OSAS to be 10%. One previous pilot study done on a Pakistani population by the authors, Taj et al [19], reported a much similar prevalence estimate of 12.4% among seminar attendees. Another study reports only the prevalence of symptoms suggestive of OSA without correlating them with the risk for OSA [18].

Studies from Asia measuring prevalence of OSAS are scarce. Indian studies done using the overnight PSG reports prevalence estimate of Sleep Disordered Breathing (SDB) (Apnea-Hypopnea Index (AHI) ≥ 5) from 13.74% to 19.5% [16]. The study also reports OSAHS (SDB with daytime hyper somnolence) to be 7.5% [16]. Data from China by Ip and colleagues [15], however, report lower prevalence of SDB (3.7%) and OSAS (2.1%). The methodology employed by these two studies, done in our neighboring Asian countries, is different from our Berlin Questionnaire based study. However, there are considerable inherent similarities within the classification of Sleep Apnea syndrome. We used Berlin Questionnaire which has 86% sensitivity for an AHI ≥ 5 and also incorporates day-time hyper-somnolence symptoms. It is, therefore, reasonable to compare results with these regional studies., Thus the point prevalence of 10% reported by our study concurs with the regional reports.

Studies done in United States and Europe that used Berlin questionnaire reported 26% prevalence estimate of High-Risk for OSAS [13,22]. Our results show lower prevalence (10%) than found in the West.

Possible reasons for this difference between prevalence of SDB and or OSAS have been attributed to cephalometeric differences [16]. These cephalometeric variations include different mandibular lengths and the anteroposterior dimensions of the nasopharynx-pharyngeal tubercle to posterior nasal spine in the skulls from the Indian subcontinent, as compared to those from other populations. This variation also point towards the possibility of an osteogenic etiology of OSAS [16]. Besides distinct genetic and environmental influences, another possible explanation perhaps is that western populations have a higher prevalence of obesity and mean BMI [25].

We report 24.9% prevalence of snoring in our population, which is similar to that reported in the Indian and Pakistani populations [16,19]. However, US population reports as high as 52–54% prevalence of snoring symptom [13,21]. This difference, once again, emphasizes the differences mentioned above.

BMI has been reported to have a linear relationship with OSAS risk in Asian [15,16] as well as Western populations [13]. Using the BMI cut-off, which is more sensitive for the Asian population [24,25] we expect a similar linear relationship between OSAS and BMI in our population as well. The difference in the prevalence of obesity, even after using a more locally sensitive cut-off, is perhaps related to lower estimate of OSAS prevalence in our population. There is significant difference of mean weight and BMI between high and low risk group (p < 0.001).

Age was also found to be an important risk factor for OSAS as has also been found by previous surveys [13,15,26]. In our sample population, almost one-in-three individuals were at high-risk among those aged 40 or more. Table 1 show mean age by risk group, there is significant difference of mean age between high and low risk group (p < 0.001). We also report an increase in the number of individuals at high risk for OSAS with increasing age till the age of 65 years. This relates to reported decline in OSAS risk after 65 years of age [13]. However, the number of respondents ≥ 65 years of age in our study was small for any significant relationship.

We also report that the snoring was significantly more common among men compared to women.(p-value 0.001) Men were also more likely to score positive for all 3 categories of the Berlin questionnaire. This conforms to the findings of a study performed on American and European subjects found that men were more likely to snore, drive while drowsy and experience breathing pauses during sleep [22]. Unlike previous studies [21,22], we did not find any gender differences for OSAS risk. Perhaps, a 2:1 proportion of males-to-females in our study population did not allow statistically significant gender differences for OSAS risk. Interestingly, a study done on a western population with analysis restricted to individuals with BMI less than 30 reported no difference in the rate of OSA risk between the two sexes [22]. Another study noted greater gender differences for OSAS risk in higher age groups [15]. These reports support our findings as 98% of our respondents have a BMI which is less than 30 and mean age of our study population is low.

Excessive day-time sleepiness and morning fatigue correlate with OSAS risk and, also, excessive daytime sleepiness is recognized as a strong indicator of OSAS [13,21]. In our sample population, these symptoms were significantly more prevalent among the high-risk group (p-value < 0.001). There is anecdotal evidence which suggests higher prevalence of fatigue and sleepiness due to longer working hours in this part of the world; hence these symptoms may not necessarily be attributable to OSAS. It may prove helpful to record the total sleeping hours of an individual along with this item of the questionnaire in further studies.

Almost eleven (10.8%) percent of our total study population reported that they had nodded off to sleep, at least once while driving. Our reported rate is similar to that reported in prior studies [13,21,22]. This is recognized as an important public health hazard and in particular, needs attention as it puts many drivers at a greater risk for road traffic accidents [12].

Hypertension was frequently reported by our sample population and was significantly more common among individuals in the high risk group. OSAS is an independent risk factor for hypertension and other cardiovascular diseases [27]. Given the high prevalence of these cardiovascular diseases, such as hypertension, in developing countries like Pakistan [28], early recognition and appropriate management of OSAS is important. This questionnaire may be used in the office setting for screening. The individuals identified as high-risk may be clinically confirmed using PSG.

Our study has certain limitations. The results are based only on data from a single, private tertiary care hospital that does not serve as representative of the whole population of the country. The study was conducted in an urban centre where risk factors associated with OSAS are expected to be high owing to the poor socio-demographic profile and lifestyle. Although the hospital from where the sample was taken is visited by people from rural areas as well, we still believe that the study setting limits the findings from being representative of the whole country. We also did not include patients who could not converse in Urdu, and this further restricts the generalization of our findings. Secondly, we used convenience sampling, and so it is not possible to quantify the error in extrapolating results to the entire population. Nevertheless, the use of the structured and validated Berlin questionnaire in our study strengthens the reliability of our results. It may be noted that the Berlin questionnaire is not particularly validated for Pakistani population. Also, a two-to-one ratio of male-to-female in our population probably overestimated the OSA-risk prevalence. The height and weight were self reported and subsequent discrepancies in the BMI had to be disregarded. We did not exclude participants with specific medical conditions, such as hypothyroidism, asthma, acromegaly, heart disease, renal disease, pregnancy, hormonal replacement therapy etc., which may further limit the strength of our analysis.

In conclusion, efforts are needed at the national and regional level and, perhaps, in other developing countries to address this problem. Considering the serious adverse health and quality-of-life consequences of OSAS, it is important to completely decipher its epidemiology. Further assessment of the prevalence of OSAS by overnight PSG along with appropriate screening tools will be meaningful. Apart from more accurate estimates, it will help evaluate the aptness and validate the screening method e.g. the Berlin questionnaire for the local population. Given the local relevance of OSAS, it is important to increase awareness among not only the general population but more importantly among the physicians of developing countries, like Pakistan, about the common clinical features, pertinent risk factors and complications of OSAS.

## Abbreviations

OSAS: Obstructive Sleep Apnea Syndrome; BMI: Body Mass Index; PSG: Polysomnography; SDB: Sleep Disordered Breathing; AHI: Apnea-hypoapnea index; OSAHS: Obstructive sleep apnea-hypopnea syndrome.

## Competing interests

The authors declare that they have no competing interests.

## Authors' contributions

FT conceived and coordinated the study. FT and ZA performed the initial literature search, analyzed the data and drafted the initial manuscript. ZA and MK were involved in collection of data and data entry. Review of initial manuscript for major intellectual content was done by MA. All authors read and approved the final manuscript.

## Pre-publication history

The pre-publication history for this paper can be accessed here:



## Supplementary Material

Additional file 1 Berlin Questionnaire (Urdu version). The *Urdu *translation of the Berlin Questionnaire which was developed using back-to-back translation of the English version of the Berlin questionnaire. *Urdu *is the national language of Pakistan. Click here for file
