# Supplementary material for: Identifying people at high risk for developing sleep apnea syndrome (SAS): a cross-sectional study in a Pakistani population
Source: BMC Neurol. 2008 Dec 17;8:50. doi: 10.1186/1471-2377-8-50 (PMC2615786; doi:10.1186/1471-2377-8-50)
Supplement: Additional file 1 — Berlin Questionnaire (Urdu version). The Urdu translation of the Berlin Questionnaire which was developed using back-to-back translation of the English version of the Berlin questionnaire. Urdu is the national language of Pakistan. [file 1471-2377-8-50-S1.pdf]

©2008. Taj et al.

برلن سوالنامہ

تاریخ .... نام سوال کنندہ .... مقام ....

جواب دہندہ کا قد (میٹرز میں) .... وزن (کلوگرام میں) .... عمر .... جنس ....

ہر سوال کے ایک درست ترین جواب کا انتخاب کریں۔

کیٹگری 1

1۔ کیا آپ خراٹے بھرتے ہیں؟

- الف ☐ جی ہاں!
- ب ☐ جی نہیں!
- ج ☐ پتا نہیں!

2۔ اگر خراٹے بھرتے ہیں تو ان کی آواز

- الف ☐ سانس لینے کی آواز سے بلند تر رہتی ہے!
- ب ☐ بولنے کی آواز کے مساوی ہوتی ہے!
- ج ☐ بولنے کی آواز سے بھی بلند تر ہوتی ہے!
- د ☐ اس قدر بلند ہوتی ہے کہ ساتھ کے کمرے میں بھی سنی جا سکتی ہے!

3۔ یہ خراٹے کب کب واقع ہوتے ہیں؟

- الف ☐ قریباً روزانہ!
- ب ☐ ہفتے میں 3 سے 4 بار!
- ج ☐ ہفتے میں 1 سے 2 بار!
- د ☐ مہینہ میں 1 سے 2 بار!
- د ☐ کبھی نہیں یا قریباً کبھی نہیں!

4۔ کیا آپ کے خراٹے کبھی دوسروں کیلئے بیزاری کا سبب بنے ہیں؟

- الف ☐ جی ہاں!
- ب ☐ جی نہیں!
- ج ☐ پتا نہیں!

5۔ کسی نے کہا ہو کہ نیند کے دوران آپ کی سانس رک بھی جاتی ہے؟

- الف ☐ قریباً ہر روز!
- ب ☐ ہفتے میں 3 سے 4 بار!
- ج ☐ ہفتے میں 1 سے 2 بار!
- د ☐ مہینے میں 1 سے 2 بار!

کیٹگری 2

6۔ مکمل نیند کے بعد بھی اکثر آپ پر تھکن کی کیفیت طاری رہتی ہے؟

- الف ☐ قریباً ہر روز!
- ب ☐ ہفتے میں 3 سے 4 بار!
- ج ☐ ہفتے میں 1 سے 2 بار!
- د ☐ مہینے میں 1 سے 2 بار!

7۔ کیا دن بھر بھی آپ پر تھکن کی کیفیت طاری رہتی ہے اور آپ خود کو تازہ دم محسوس نہیں کرتے؟

- الف ☐ قریباً ہر روز!
- ب ☐ ہفتے میں 3 سے 4 بار!
- ج ☐ ہفتے میں 1 سے 2 بار!
- د ☐ مہینے میں 1 سے 2 بار!

8۔ کیا گاڑی چلاتے ہوئے کبھی آپ نے خود پر اونگھ یا نیند کا غلبہ محسوس کیا ہے؟

- الف ☐ ہاں!
- ب ☐ نہیں!

9۔ اگر ہاں تو

- الف ☐ قریباً ہر روز!
- ب ☐ ہفتے میں 3 سے 4 بار!
- ج ☐ ہفتے میں 1 سے 2 بار!
- د ☐ مہینے میں 1 سے 2 بار!

کیٹگری 3

10۔ آپ کا بلڈ پریشر زیادہ تو نہیں رہتا؟

- الف ☐ ہاں!
- ب ☐ نہیں!
- ج ☐ پتا نہیں!

©2008. Taj et al.
